# Mathematical metacognition and computational thinking in adolescents: the roles of cognitive flexibility and grade level

**DOI:** 10.3389/fpsyg.2026.1806297

**Published:** 2026-06-16

**Authors:** Mehmet Bars

**Affiliations:** Department of Basic Education, Ziya Gökalp Faculty of Education, Dicle University, Diyarbakır, Türkiye

**Keywords:** adolescence, cognitive flexibility, computational thinking, mathematical metacognition, moderated mediation analysis

## Abstract

**Background:**

Computational thinking is a key higher-order cognitive skill in adolescence, extending beyond computer science to mathematical problem solving and complex reasoning. Although mathematical metacognition has been associated with computational thinking, the mechanisms underlying this relationship remain unclear. Grounded in Flavell’s metacognitive theory and Wing’s framework, this study examines the mediating role of cognitive flexibility and the moderating role of grade level in this association among high school students.

**Method:**

A cross-sectional study was conducted with 467 high school students (M_*age*_ = 16.05, *SD* = 1.20, 57.2% female) from grades 9–12 in Diyarbakır, Türkiye. Participants completed validated self-report measures of mathematical metacognition, cognitive flexibility, and computational thinking. The proposed moderated mediation model was tested using Hayes’ PROCESS macro (Model 14).

**Results:**

Mathematical metacognition was positively associated with computational thinking. Mediation analyses showed that cognitive flexibility partially mediated this relationship, indicating both direct and indirect associations between metacognitive regulation and computational thinking. In addition, the interaction between cognitive flexibility and grade level was significant, indicating that this association varied across grade levels. Simple slope analyses showed that the positive association between cognitive flexibility and computational thinking strengthened at higher grade levels. The index of moderated mediation was significant (IMM = 0.065, 95% CI [0.012, 0.141]), indicating that the indirect association varied systematically across grade levels.

**Conclusion:**

The findings suggest that cognitive flexibility may represent an important mechanism underlying the association between mathematical metacognition and computational thinking within a developmental framework. By demonstrating that this association becomes stronger across grade levels, the findings support the value of integrating metacognitive and flexibility-oriented practices in secondary education to promote computational thinking.

## Graphical Abstract

**Figure:**
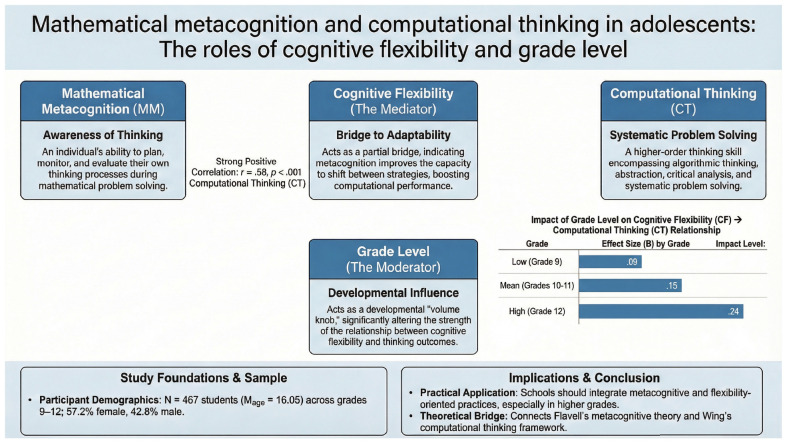
Proposed moderated mediation model showing that cognitive flexibility mediates the relationship between mathematical metacognition and computational thinking, while grade level moderates the association between cognitive flexibility and computational thinking. The indirect effect of mathematical metacognition on computational thinking becomes stronger at higher grade levels.

## Introduction

Computational thinking is defined as a core way of thinking that enables individuals to solve complex problems systematically, encompassing cognitive skills such as algorithmic thinking, abstraction, critical thinking, and creative problem solving (Shute et al., 2017; Wing, 2006). This mode of thinking is not limited to computer science or programming contexts; it is also closely related to mathematical problem solving, coping with everyday cognitive challenges, and academic learning processes (Grover and Pea, 2013). In recent years, computational thinking has increasingly been viewed as a cross-disciplinary skill and has been linked not only to students’ technological competencies but also to their performance on tasks that require cognitive flexibility and transfer (Rich et al., 2017; Shute et al., 2017; Wing, 2011).

Particularly during adolescence, the development of computational thinking skills is regarded as a key factor supporting students’ learning performance, problem-solving abilities, and higher-order cognitive functions. Indeed, higher levels of computational thinking during this period are associated with more effective academic task performance, greater adaptability to new situations, and improved management of complex cognitive demands (Diamond, 2013; Korkmaz et al., 2017). For this reason, computational thinking is accepted as an important indicator of individual cognitive development and academic adjustment, while adolescence is seen as a critical period for the development of this skill (Diamond, 2013; Grover and Pea, 2013; Shute et al., 2017). Consequently, it becomes increasingly important to examine computational thinking not only through its outcomes but also through the cognitive processes that may support its development. In this context, investigating the factors that influence high school students’ computational thinking skills and explaining the underlying psychological mechanisms is considered a key research need.

Among these, one of the cognitive structures that influences the development of computational thinking is mathematical metacognition. Mathematical metacognition is defined as an individual’s awareness of their own thinking processes during mathematical problem solving, as well as their ability to plan, monitor, and evaluate these processes (Flavell, 1979). Students’ levels of mathematical metacognition directly affect the effectiveness of the strategies they use while solving problems, their capacity to recognize errors, and their ability to revise solution paths when necessary (Flavell, 1979; Schoenfeld, 2013; Schraw and Dennison, 1994). From this perspective, mathematical metacognition is viewed not only as a contributor to cognitive performance but also as a regulatory structure associated with the flexible and adaptive use of cognitive processes (Schraw and Dennison, 1994; Zohar and Barzilai, 2013).

Today, rising academic expectations and increasingly complex curricula require students not only to possess knowledge but also to use that knowledge in a conscious and self-regulated manner (Bransford et al., 2000; OECD, 2019). As a result, mathematical metacognition can be considered an increasingly important cognitive resource for high school students. Previous research has documented significant associations between mathematical metacognition and academic achievement, problem-solving performance, and higher-order thinking skills (Schoenfeld, 2014; Zohar and Barzilai, 2013). However, how mathematical metacognition is related to computational thinking skills, and through which mechanisms this relationship operates, has not yet been sufficiently clarified. This suggests that the relationship between mathematical metacognition and computational thinking may involve both direct and indirect mechanisms.

Previous research suggests that the associations between cognitive structures and behavioral outcomes often operate through indirect pathways with certain psychological mechanisms playing a mediating role in this process (Baron and Kenny, 1986; Kovan, 2026). Within this framework, cognitive flexibility is defined as an individual’s capacity to adapt to changing conditions, shift between different problem-solving strategies, and develop alternative perspectives. In other words, cognitive flexibility is considered a core executive function that allows individuals to reorganize their thinking strategies according to situational demands (Diamond, 2013; Ionescu, 2012). Accordingly, cognitive flexibility is widely regarded as one of the key determinants of effective cognitive performance, particularly in complex, multi-step problem-solving processes.

Given that mathematical metacognition involves the conscious regulation of problem-solving processes and strategic decision making, it may also be associated with higher levels of cognitive flexibility (Diamond, 2013; Ionescu, 2012; Zohar and Barzilai, 2013). Similarly, higher cognitive flexibility may be associated with more effective use of computational thinking skills (Rich et al., 2017; Shute et al., 2017). However, a review of the existing literature indicates that studies directly examining the mediating role of cognitive flexibility in the relationship between mathematical metacognition and computational thinking are limited. This gap highlights the potential importance of cognitive flexibility as a psychological mechanism underlying the relationship between these two constructs.

However, the effects of cognitive processes on individuals do not emerge in the same way across all developmental stages. During the high school years, students face increasing academic responsibilities, more intensive and complex learning tasks, and rising cognitive expectations as they progress through grade levels. This process contributes to both quantitative and qualitative differences in cognitive skills (OECD, 2019; Steinberg, 2014). Within this framework, it can be expected that the contribution of higher-order cognitive skills, such as cognitive flexibility, to computational thinking may vary by grade level. Nevertheless, existing studies have generally examined the relationships among mathematical metacognition, cognitive flexibility, and computational thinking without sufficiently considering how these associations vary across grade levels. As students progress through secondary education, increasing academic demands and performance-oriented learning environments may shape how effectively they use cognitive flexibility during problem solving (OECD, 2019; Shute et al., 2017). Accordingly, grade level may reflect not only chronological progression but also differences in students’ academic experiences and cognitive demands.

### Mathematical metacognition and computational thinking

A substantial body of research shows a close relationship between individuals’ cognitive and metacognitive characteristics and their higher-order thinking skills. In particular, metacognitive regulation processes are consistently associated with problem-solving performance and success in complex cognitive tasks (Flavell, 1979; Schraw and Dennison, 1994; Zimmerman, 2002; Zohar and Barzilai, 2013). Mathematical metacognition is considered an important cognitive construct that refers to an individual’s conscious regulation of their cognitive processes during mathematical problem solving through planning, monitoring, and evaluation. This construct is associated with greater cognitive awareness, which may help students make more deliberate and strategic decisions during problem solving (Flavell, 1979; Schoenfeld, 2013). Moreover, mathematical metacognition is also associated with students’ ability not only to arrive at correct solutions, but also to manage the solution process itself in a flexible and strategic manner (Zohar and Barzilai, 2013).

Computational thinking is commonly defined as a higher-order thinking skill involving the integrated use of cognitive processes such as algorithmic thinking, abstraction, critical analysis, and creative problem solving (Korkmaz et al., 2017; Wing, 2006). From this perspective, computational thinking is closely associated with an individual’s capacity to structure problems, systematically plan solution pathways, and evaluate the problem-solving process itself (Grover and Pea, 2013; Shute et al., 2017). Because these processes involve monitoring and reorganizing cognitive activity when necessary, computational thinking has a strong theoretical connection with metacognitive regulation. Indeed, greater metacognitive awareness in mathematical problem-solving contexts may be associated with more effective use of cognitive resources and to develop more flexible and adaptive strategies throughout the problem-solving process (Schoenfeld, 2013; Schraw and Dennison, 1994; Zohar and Barzilai, 2013). Taken together, these findings point to a theoretically meaningful relationship between mathematical metacognition and computational thinking skills.

Metacognitive theories suggest that an individual’s capacity to monitor and regulate their own cognitive processes is closely associated with complex problem solving and higher-order thinking skills. According to this perspective, individuals with stronger metacognitive regulation skills may be more likely to generate alternative strategies during problem solving, detect their errors more easily, and restructure solution paths when necessary (Flavell, 1979; Schoenfeld, 2013; Zimmerman, 2002). Considering the algorithmic structuring, abstraction, and systematic solution generation involved in computational thinking, mathematical metacognition may represent an important cognitive resource associated with computational thinking. Indeed, several studies indicate that students with higher levels of mathematical metacognition tend to report stronger problem-solving performance and more advanced higher-order cognitive skills (Shute et al., 2017; Wing, 2006, 2011; Zhang et al., 2024).

Moreover, studies that directly examine the relationship between mathematical metacognition and computational thinking are limited, and existing research tends to address these two constructs in separate contexts (Grover and Pea, 2013; Shute et al., 2017; Zohar and Barzilai, 2013). In addition, the mechanisms underlying the relationship between mathematical metacognition and computational thinking have not yet been clearly explained. Within this framework, it can be argued that the relationship between mathematical metacognition and computational thinking may operate not only directly but also indirectly through cognitive regulation processes. Building on these considerations, examining whether mathematical metacognition is associated with computational thinking skills may contribute to a clearer understanding of the relationship between these two important cognitive constructs. Accordingly, the following hypothesis is proposed in the present study:

*H1*: Mathematical metacognition positively and significantly predicts computational thinking.

### Cognitive flexibility as a mediator

In the process of coping with complex cognitive tasks, not only the presence of cognitive skills but also how these skills are regulated and applied is closely associated with effective performance in complex cognitive tasks (Bransford et al., 2000; Zimmerman, 2002; Zohar and Barzilai, 2013). For this reason, cognitive flexibility can be viewed as a functional outcome of metacognitive regulation. The effective use of metacognitive processes involves recognizing not only which strategy to use, but also when and under what conditions a strategy should be changed (Flavell, 1979; Schoenfeld, 2013, 2014). Within this context, cognitive flexibility is understood as the capacity to adapt to changing conditions, shift between different problem-solving strategies, and effectively evaluate alternative solution paths, suggesting that it may represent an important mechanism within this relationship. From this perspective, cognitive flexibility is considered a dynamic indicator of cognitive adaptability (Schoenfeld, 2013; Schraw and Dennison, 1994). Accordingly, the present study proposes that cognitive flexibility may function as a mediating variable in the relationship between mathematical metacognition and computational thinking.

Although these constructs are conceptually related, their theoretical boundaries should be distinguished clearly. Mathematical metacognition refers to the awareness and regulation of cognitive processes in mathematical contexts (Flavell, 1979). Cognitive flexibility refers to the ability to shift adaptively between cognitive strategies (Diamond, 2013; Miyake et al., 2000), whereas computational thinking emphasizes structured problem decomposition and algorithmic reasoning (Wing, 2006). Prior psychometric research also supports the distinction among these constructs (Miyake et al., 2000; Schraw and Dennison, 1994; Wing, 2006). The development of cognitive flexibility is, in fact, closely linked to metacognitive regulation processes. According to Flavell’s metacognitive theory, individuals have the capacity to monitor, evaluate, and reorganize their own cognitive processes when necessary. These processes are thought to support strategy selection and flexible strategy use during problem solving (Flavell, 1979). Within this model, metacognitive knowledge and metacognitive regulation are defined as the core mechanisms that guide an individual’s cognitive behavior. From this perspective, mathematical metacognition is associated with individuals’ ability to recognize effective strategies during problem solving, monitor their errors, and adjust their cognitive approach when needed (Bars, 2022, 2026; Flavell, 1979; Schraw and Dennison, 1994). As these explanations suggest, such processes may contribute to the development of cognitive flexibility rather than cognitive rigidity. Accordingly, it can be expected that these metacognitive regulation processes may be associated with higher levels of cognitive flexibility.

On the other hand, cognitive flexibility is also closely related to the complex cognitive processes involved in computational thinking. As noted earlier, computational thinking is a complex form of thinking that involves decomposing problems, making abstractions, developing algorithmic solution pathways, and evaluating the solution process (Grover and Pea, 2013; Wing, 2006). Each of these processes involves shifting between different cognitive strategies during problem solving and, when necessary, to restructure their solution approach. In such contexts, it may be beneficial for individuals to avoid relying on a single solution path, but instead are able to try alternative strategies and revise their approach when needed (Ionescu, 2012; Zohar and Barzilai, 2013). Within this framework, higher cognitive flexibility may be associated with more effective use of computational thinking skills in a more effective and functional manner. Accordingly, cognitive flexibility can be considered a fundamental cognitive resource that may support the effective application of computational thinking.

Some studies in the literature indicate that metacognitive skills support the development of cognitive flexibility (Flavell, 1979; Ionescu, 2012), and that cognitive flexibility is positively associated with problem-solving performance and higher-order thinking skills (Miyake et al., 2000; Schoenfeld, 2014). However, the mechanisms underlying the relationship between mathematical metacognition and computational thinking have yet to be adequately clarified. Existing research tends to link mathematical metacognition and computational thinking directly (Grover and Pea, 2013; Shute et al., 2017), without examining in detail the cognitive processes underlying this relationship. This gap suggests that cognitive flexibility may serve as a key mediating mechanism that may help account for the association between mathematical metacognition and computational thinking.

Within this framework, cognitive flexibility can be considered an important psychological mechanism for explaining the relationship between mathematical metacognition and computational thinking. Specifically, by supporting individuals’ conscious regulation of their cognitive processes, mathematical metacognition may be associated with greater cognitive flexibility, which in turn may relate to more effective use of computational thinking skills. In other words, mathematical metacognition may be linked to more adaptive forms of computational thinking through cognitive flexibility. Accordingly, the present study proposes that cognitive flexibility plays a mediating role in the relationship between mathematical metacognition and computational thinking. On this basis, the following hypothesis is proposed:

*H2*: Cognitive flexibility mediates the relationship between mathematical metacognition and computational thinking.

### Grade level as a moderator

Previous research examining the relationship between cognitive flexibility and higher-order cognitive skills has often treated grade level merely as a control variable (Best and Miller, 2010; Diamond, 2013). This approach considers grade level primarily as a demographic characteristic, while often overlooking its potential role in cognitive development, academic experience, and learning context. However, studies in developmental and educational psychology suggest that the associations involving cognitive processes may vary depending on students’ level of academic experience and stage of cognitive maturation (Cherukunnath and Singh, 2022; Steinberg, 2014). For this reason, the present study assumes that the association between cognitive flexibility and computational thinking may differ across high school grade levels.

During the course of high school education, students face increasing academic expectations, more complex and demanding problem-solving tasks, and a growing cognitive load as they advance through grade levels (Chang and Yang, 2010; Ma, 2025). In upper grades in particular, the abstract concepts and multi-step problem-solving processes encountered in subjects such as mathematics and science may encourage students to use their cognitive strategies in more flexible ways (Rio and Protacio, 2025). In this sense, cognitive flexibility may become increasingly important for the effective use of computational thinking skills. Accordingly, cognitive flexibility can be regarded as an increasingly important academic resource associated with computational thinking (Avcı, 2025).

From a developmental perspective, cognitive control and metacognitive regulation skills develop gradually throughout adolescence. Students in lower grade levels may be more likely to rely on a single strategy during problem-solving processes, whereas students in higher grade levels become more capable of generating alternative solution paths, detecting their errors more quickly, and restructuring their cognitive approaches in response to situational demands (Badolo et al., 2025). This developmental progression may strengthen the association between cognitive flexibility and higher-order cognitive outcomes (Honra and Monterola, 2026). These developmental differences suggest that the association between cognitive flexibility and computational thinking may become stronger as grade level increases.

Furthermore, grade level is also associated with differences in cognitive development but also students’ academic self-efficacy beliefs, problem-solving experiences, and learning contexts (Ramos-Salazar and Hayward, 2019). Upper-grade students, drawing on the academic experiences accumulated over previous years, may use cognitive flexibility more strategically, which may in turn be associated with stronger computational thinking skills. These students tend to have a broader repertoire of problem-solving strategies and are able to use cognitive flexibility more deliberately when faced with challenges (Best and Miller, 2010; Schoenfeld, 2014; Shute et al., 2017). By contrast, at lower grade levels, where cognitive flexibility has not yet fully matured, the association between this skill and computational thinking may be weaker.

In light of these findings and explanations, the relationship between cognitive flexibility and computational thinking is expected to differ across high school grade levels. Put differently, while the association between cognitive flexibility and computational thinking is expected to be present for all high school students, this effect is anticipated to be stronger at higher grade levels. This perspective suggests that grade level should not be viewed merely as a variable to be controlled, but rather as an important contextual factor that shapes the relationship between cognitive processes and computational thinking. Accordingly, the present study examines whether the relationship between cognitive flexibility, positioned as a mediator in the link between mathematical metacognition and computational thinking, and computational thinking differs by high school grade level. Furthermore, the study investigates whether the indirect association between mathematical metacognition and computational thinking through cognitive flexibility varies as a function of grade level. This approach aims to clarify how the relationship between mathematical metacognition and computational thinking varies within a developmental context. On this basis, the following hypothesis is proposed:

*H3*: (a) Grade level moderates the association between cognitive flexibility and computational thinking such that (b) the positive relationship between cognitive flexibility and computational thinking becomes stronger as grade level increases.

**FIGURE 1:**
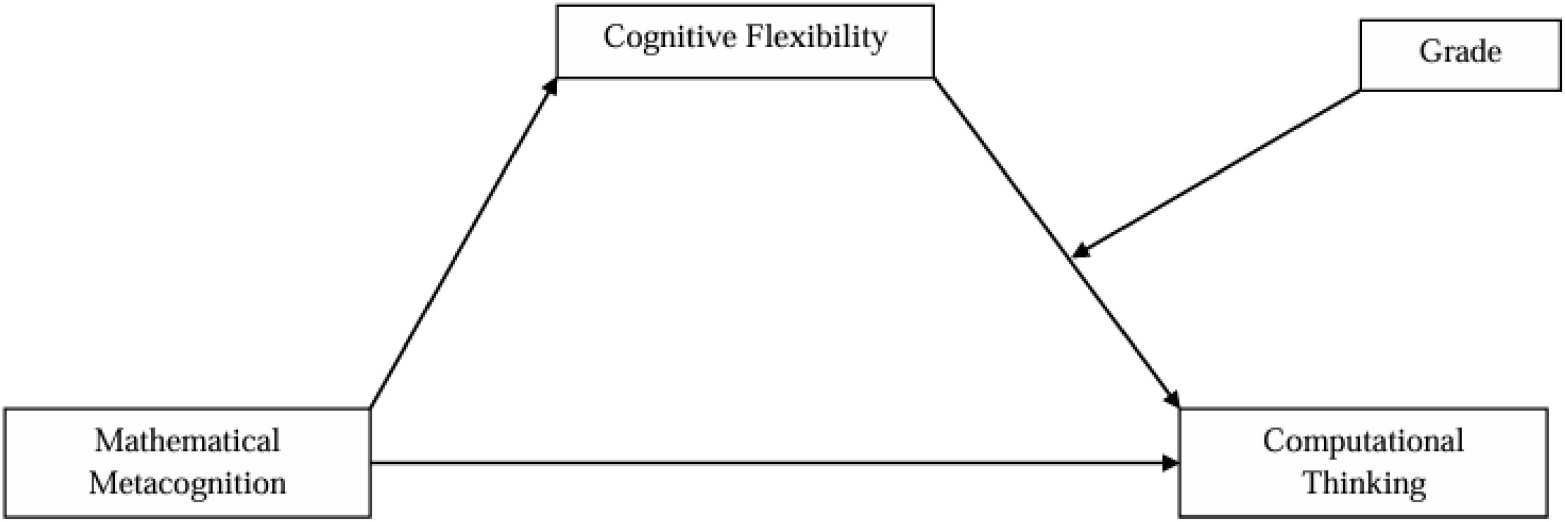
The proposed moderated mediation model.

Accordingly, this study aims to test a moderated mediation model that examines the mediating role of cognitive flexibility in the relationship between mathematical metacognition and computational thinking among high school students, as well as the moderating effect of grade level on this indirect relationship. This model provides a framework for understanding how the relationships between cognitive processes and higher-order thinking skills may differ within the developmental context of adolescence. Within this scope, the proposed research model is presented in Figure 1.

### Present study

This study aims to contribute to the literature in three main ways by examining the relationship between mathematical metacognition and computational thinking among high school students, with particular attention to cognitive flexibility and grade level. First, rather than focusing solely on the direct relationship between mathematical metacognition and computational thinking, the study offers a process-oriented perspective by examining the role of cognitive flexibility in this relationship. Second, by conceptualizing cognitive flexibility as a potential psychological mechanism, the study further develops the theoretical connection between metacognitive regulation and higher-order thinking skills. Third, instead of treating grade level merely as a demographic variable, the study conceptualizes it as a developmental context that may shape the association between cognitive flexibility and computational thinking and tests a moderated mediation model accordingly. From this perspective, the study contributes to understanding how cognitive and educational processes interact within a developmental framework and offers both theoretical and practical implications for supporting computational thinking among high school students.

## Materials and methods

### Participants and procedure

The present study employed a cross-sectional design and was conducted with high school students in Diyarbakır, Türkiye. Data were collected during the 2025–2026 academic year using a convenience-based cluster sampling approach. This sampling strategy was adopted to ensure practical feasibility within the school context while allowing access to students from different grade levels. A total of 467 valid questionnaires were included in the final analyses after excluding incomplete or invalid responses. The final sample consisted of 200 male students (42.8%) and 267 female students (57.2%). Participants were drawn from grades 9 through 12, including 90 ninth-grade students (19.3%), 65 tenth-grade students (13.9%), 121 eleventh-grade students (25.9%), and 191 twelfth-grade students (40.9%). The age of participants ranged from 13 to 18 years (M = 16.05, *SD* = 1.20). An a priori power analysis conducted using GPower (v.3.1) indicated that a minimum sample size of 353 participants was required to detect small-to-medium effects (*f*^2^ = 0.03) with α = 0.05 and statistical power (1 - β) = 0.90 (Faul et al., 2009). The final sample of 467 students therefore provided sufficient statistical power for the proposed analyses.

Prior to data collection, official permission was obtained from the relevant school administrations. Moreover, ethical approval was granted by the Ethics Committee of the affiliated university (Dicle University Social and Human Sciences Ethics Committee: Decision No. 551, 15/12/2025) before the initiation of the study. All research procedures were conducted in accordance with ethical principles for research involving human participants. Participation was voluntary, and students were informed about the purpose of the study, the anonymity of their responses, and their right to withdraw at any time without penalty. Given that the participants were minors, informed consent was also obtained from their parents or legal guardians prior to data collection. In addition, informed assent was obtained from all participating students before questionnaire administration. The questionnaires were administered in classroom settings during regular school hours under the supervision of a trained researcher and a classroom teacher. Participants completed the paper-and-pencil questionnaires individually, following standardized instructions emphasizing honest and independent responses. The survey required approximately 20–25 min to complete.

### Measures

#### Mathematical metacognition

Mathematical metacognition was assessed using the Turkish version of the Metacognition in Mathematics Scale, originally developed by Fung and Leung (2017) and adapted into Turkish by Bars (2022). The scale consists of 16 items rated on a 7-point Likert scale (1 = strongly disagree, 7 = strongly agree) across four factors: prediction, planning, monitoring, and evaluation. Higher scores indicate higher levels of mathematical metacognition. In the present study, the scale demonstrated high internal consistency (Cronbach’s α = 0.87). A confirmatory factor analysis (CFA) supported the four-factor structure with acceptable model fit indices (χ^2^/df = 4.36, CFI = 0.86, TLI = 0.81, RMSEA = 0.08). Although some incremental fit indices fell slightly below conventional cutoffs, the overall pattern of fit and reliability supported the adequacy of the measurement model for the present study (Kline, 2023; Marsh et al., 2004).

#### Cognitive flexibility

Cognitive flexibility was measured using the Cognitive Flexibility Scale developed by Çelikkaleli (2014). The scale consists of 11 items rated on a 5-point Likert scale (1 = strongly disagree, 5 = strongly agree) and assesses individuals’ capacity to adapt to changing situations and flexibly regulate cognitive strategies. Higher scores reflect higher levels of cognitive flexibility. In the present study, the scale showed excellent internal consistency (Cronbach’s α = 0.91). CFA results supported the unidimensional structure with acceptable model fit indices (χ^2^/df = 4.02, GFI = 0.93, CFI = 0.82, RMSEA = 0.08). Collectively, the fit indices and reliability coefficients supported the use of the scale in the present sample.

#### Computational thinking

Computational thinking was assessed using the Computational Thinking Skills Scale developed by Korkmaz et al. (2017). The scale consists of 29 items rated on a 5-point Likert scale (1 = strongly disagree, 5 = strongly agree) across five dimensions: creativity, algorithmic thinking, collaboration, critical thinking, and problem solving. Higher scores indicate stronger computational thinking skills. In the present study, the scale demonstrated good internal consistency (Cronbach’s α = 0.84). CFA supported the five-factor structure with acceptable fit indices (χ^2^/df = 2.73, CFI = 0.84, TLI = 0.83, RMSEA = 0.06). Although computational thinking may also be assessed through performance-based tasks, self-report instruments are commonly used in large-scale educational research examining perceived computational competencies and their cognitive correlates. The limitations associated with self-report assessment are addressed in the Limitations section.

### Convergent and discriminant validity

To supplement the CFA results, standardized factor loadings, composite reliability (CR), and average variance extracted (AVE) were computed for each scale. All factor loadings were statistically significant (*p* < 0.001) and ranged from 0.50 to 0.84. CR values ranged from 0.79 to 0.93, exceeding the recommended 0.70 threshold (Fornell and Larcker, 1981). Most AVE values approached or exceeded the recommended 0.50 criterion, although several computational thinking subscales showed marginally lower values (0.45–0.49). Discriminant validity was generally supported using the Fornell–Larcker criterion, as the square root of each construct’s AVE exceeded the corresponding inter-construct correlations (see Table 1).

**TABLE 1:** CFA results: standardized factor loadings, composite reliability, and average variance extracted.

Scale	Factor	*n*	Loadings (range)	CR	AVE
Mathematical Metacognition	Prediction	4	0.56–0.71	0.79	0.48
	Planning	4	0.60–0.73	0.81	0.51
	Monitoring	4	0.58–0.74	0.82	0.53
	Evaluation	4	0.55–0.72	0.80	0.50
Cognitive flexibility	–	11	0.64–0.84	0.93	0.57
Computational thinking	Creativity	6	0.52–0.70	0.82	0.47
	Algorithmic thinking	6	0.54–0.71	0.83	0.49
	Collaboration	6	0.50–0.68	0.80	0.45
	Critical thinking	5	0.55–0.72	0.81	0.48
	Problem solving	6	0.53–0.72	0.82	0.47

CR, composite reliability; AVE, average variance extracted. All factor loadings were statistically significant at *p* < 0.001. CR > 0.70 (Fornell and Larcker, 1981). AVE slightly below the recommended 0.50 threshold; discriminant validity was nonetheless supported as √AVE (0.68, 0.75, and 0.68 for mathematical metacognition, cognitive flexibility, and computational thinking, respectively) exceeded all inter-construct correlations (0.32, 0.58, 0.28).

### Data analysis

All statistical analyses were conducted using IBM SPSS (v.23) and AMOS (v.23). Initially, SPSS was used to screen the data, compute descriptive statistics, and examine zero-order correlations among the study variables. Prior to hypothesis testing, missing data patterns and assumptions of univariate normality were inspected. Skewness and kurtosis values for all variables fell within ±1.5, suggesting acceptable univariate normality for applied research (Tabachnick and Fidell, 2019).

To assess the construct validity of the measurement instruments, confirmatory factor analyses (CFA) were conducted separately for each scale using AMOS. Model fit was evaluated using multiple fit indices, including the chi-square statistic (χ^2^), the ratio of chi-square to degrees of freedom (χ^2^/df), the Comparative Fit Index (CFI), the Tucker–Lewis Index (TLI), and the Root Mean Square Error of Approximation (RMSEA). Following commonly accepted guidelines, model fit was considered acceptable when χ^2^/df values were below 5.0, CFI and TLI values were close to or above 0.90, and RMSEA values were below. 008 (Hu and Bentler, 1999; Kline, 2023).

After establishing measurement validity, hypothesis testing was conducted using Hayes’ PROCESS macro (v.4.2), with Model 14 specifying mathematical metacognition as the independent variable, computational thinking as the dependent variable, cognitive flexibility as the mediator, and grade level as the moderator of the M→Y path. Although CFA was conducted to confirm the factorial validity of the scales, subsequent analyses were performed using composite scores, consistent with common practice in educational and developmental research employing conditional process analysis (Hayes, 2018; Preacher and Hayes, 2008). PROCESS Model 14 was selected because it allows the simultaneous estimation of direct, indirect, and moderated associations using bootstrapped confidence intervals (CIs) within a regression-based framework. Future research could extend these findings using latent-variable SEM approaches.

Indirect effects were tested using a bias-corrected bootstrapping procedure with 5,000 resamples. An indirect or conditional effect was considered statistically significant when the 95% bootstrap CI did not include zero (Hayes, 2018; Preacher and Hayes, 2008). Grade level was treated as a quasi-continuous moderator representing ordinal developmental progression across secondary education (grades 9–12). This approach is commonly used when theoretical expectations emphasize monotonic developmental change rather than categorical group differences (Whisman and McClelland, 2005). The decision is also consistent with developmental frameworks emphasizing the progressive maturation of executive functions, including cognitive flexibility, across adolescence (Best and Miller, 2010; Diamond, 2013). Nevertheless, future studies may employ multi-group SEM or non-linear approaches to further examine potential grade-specific patterns. All continuous variables were mean-centered prior to the creation of interaction terms to reduce multicollinearity. Statistical significance was evaluated at *p* < 0.05.

## Results

### Common method bias and multicollinearity

As all variables were assessed using self-report questionnaires, Harman’s single-factor test was conducted to examine the potential influence of common method bias. The results indicated that multiple factors had eigenvalues greater than 1, and the first factor accounted for approximately 25% of the total variance, which is below the recommended threshold of 40% (Podsakoff et al., 2003). Although Harman’s test is one commonly applied check, its limitations as a sole diagnostic tool are acknowledged (Podsakoff et al., 2003). Additional confidence in the discriminant structure of the data is provided by the CFA and AVE-based discriminant validity analyses reported.

Overall, these diagnostics suggest that common method bias is unlikely to have substantially distorted the observed relationships, though future research incorporating multiple informants or objective performance measures would further strengthen this conclusion. In addition, variance inflation factor (VIF) values were approximately 1 and tolerance values were close to 1, indicating that multicollinearity was not a concern (Tabachnick and Fidell, 2019). All condition index values were below 30, and no problematic variance proportions were observed across predictors.

### Preliminary analysis

Descriptive statistics and zero-order Pearson correlation coefficients for the study variables are presented in Table 2. Mathematical metacognition was positively and significantly associated with computational thinking (*r* = 0.58, *p* < 0.001) and cognitive flexibility (*r* = 0.32, *p* < 0.001). Cognitive flexibility also showed a significant positive association with computational thinking (*r* = 0.28, *p* < 0.001). These results provide preliminary support for the proposed mediating role of cognitive flexibility.

**TABLE 2:** Descriptive statistics and correlations.

Variables	M	*SD*	ω	λ 6	1	2	3
1. Mathematical metacognition	73.98	15.77	0.86	0.89	–		
2. Cognitive flexibility	39.28	3.82	0.89	0.92	0.32***	–	
3. Computational thinking	105.13	14.72	0.94	0.93	0.58***	0.28***	–

ω = McDonald’s omega, λ6 = Guttman’s lambda 6; ***p < 0.001.

### Testing the mediation effect

The findings presented in Table 3 indicate that mathematical metacognition significantly and positively predicts cognitive flexibility (β = 0.34, *p* < 0.001). Mathematical metacognition also directly and significantly predicts computational thinking, supporting H_1_. Model-level effect size estimates indicated a medium-to-large effect size for cognitive flexibility (*f*^2^ = 0.27, *R*^2^ = 0.21) and a large effect size for computational thinking (*f*^2^ = 0.75, *R*^2^ = 0.43), based on Cohen’s (1988) benchmarks (0.02 = small, 0.15 = medium, 0.35 = large). The indirect association between mathematical metacognition and computational thinking through cognitive flexibility was statistically significant, as indicated by the 95% bootstrap CI excluding zero. Although the direct association between mathematical metacognition and computational thinking remained significant after including cognitive flexibility in the model, its magnitude decreased, suggesting partial mediation. Thus, H2 was supported.

**TABLE 3:** Mediating effect of cognitive flexibility between mathematical metacognition and computational thinking.

Variables	β	SE	*p*	*R* ^2^	*f* ^2^	95% CI
Dependent: cognitive flexibility						
Constant	–1.33	0.25	0.00	0.21	0.27	[–1.78, –0.89]
Mathematical metacognition	0.34	0.09	0.00			[0.26, 0.60]
Dependent: computational thinking						
Constant	1.10	0.24	0.00	0.43	0.75	[0.68, 1.52]
Mathematical metacognition	0.46	0.10	0.00			[0.31, 0.60]
Cognitive flexibility (M)	0.31	0.11	0.00			[0.14, 0.48]
Grade (W)	-0.09	0.10	0.29			[–0.07, 0.20]
Interaction term (M × W)	0.19	0.10	0.04			[0.04, 0.35]

β = standardized regression coefficient; f^2^ = model-level effect size calculated as R^2^/(1 - R^2^). According to Cohen’s (1988) benchmarks, 0.02 = small, 0.15 = medium, and 0.35 = large. Bootstrapped 95% CIs are based on 5,000 resamples.

### Testing the moderated mediation effect

In the second stage of the model, the moderating role of grade level was examined. Grade level did not have a significant direct effect on computational thinking (β = -0.09, *p* > 0.05). However, the interaction term between cognitive flexibility and grade level was positive and statistically significant (β = 0.19, *p* < 0.05), indicating that grade level moderates the association between cognitive flexibility and computational thinking. Therefore, H_3a_ was supported (cf. Table 4). To further examine whether grade level moderated the indirect pathway, the index of moderated mediation (IMM) was computed following Hayes (2018). The IMM was 0.065 (SE = 0.033, 95% CI [0.012, 0.141]). Because the confidence interval excluded zero, the indirect association between mathematical metacognition and computational thinking through cognitive flexibility was found to vary across grade levels, supporting the presence of moderated mediation.

**TABLE 4:** Conditional indirect effects of mathematical metacognition on computational thinking through cognitive flexibility.

Moderator variable	B	SE	95% CI
Grade (−1 SD)	0.09	0.04	[0.02, 0.18]
Grade (M)	0.15	0.05	[0.06, 0.26]
Grade (+1 SD)	0.24	0.07	[0.12, 0.38]
Index of moderated mediation (IMM)	0.065	0.033	[0.012, 0.141]

IMM, index of moderated mediation.

**FIGURE 2:**
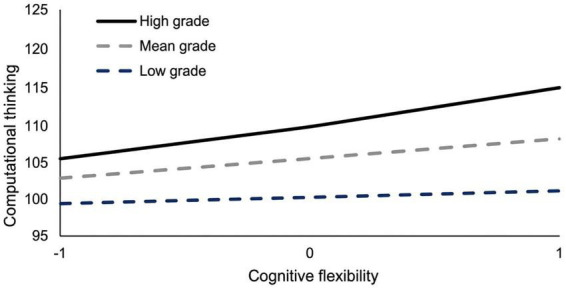
Simple slopes illustrating the relationship between cognitive flexibility and computational thinking across low (–1 SD), mean, and high (+1 SD) levels of grade.

To facilitate interpretation of the interaction effect, a simple slope analysis was conducted at low (-1 *SD*), mean, and high (+1 *SD*) levels of grade level. As illustrated in Figure 2, cognitive flexibility was positively associated with computational thinking across all grade levels, with the strength of this association increasing at higher grade levels. The line representing higher grade levels is steeper than that for lower grade levels, indicating that the association between cognitive flexibility and computational thinking becomes stronger as students advance through secondary school. Table 4 shows that the conditional indirect effects were significant at all three levels of grade level and increased systematically from.09 (95% CI [0.02, 0.18]) at one *SD* below the mean to 0.24 (95% CI [0.12, 0.38]) at one *SD* above the mean, supporting the moderated mediation pattern. These findings suggest that the positive association between cognitive flexibility and computational thinking becomes stronger at higher grade levels, supporting H_3b_.

## Discussion

The findings of this study indicate that the relationships among mathematical metacognition, cognitive flexibility, and computational thinking vary across grade levels and operate through interconnected cognitive mechanisms. The results show that mathematical metacognition is associated with computational thinking both directly and indirectly through cognitive flexibility. Furthermore, the strength of this indirect relationship differs across grade levels, suggesting that these associations may change throughout adolescence.

First, the finding that mathematical metacognition significantly and positively predicts computational thinking is consistent with previous research emphasizing the central role of metacognitive regulation processes in complex problem-solving skills (Bars, 2022; Rio and Protacio, 2025; Schoenfeld, 2014). This consistency may reflect the association between mathematical metacognition and heightened awareness of the problem-solving process, which may allow students to more consciously regulate when, how, and which strategies are used. In doing so, it is associated with the algorithmic, systematic, and planned thinking processes involved in computational thinking. Stated differently, mathematical metacognition is associated with greater capacity to plan, monitor, and evaluate problem-solving activities, allowing students to approach problems more strategically (Güner and Erbay, 2021; Tachie, 2019). This finding is consistent with Flavell’s metacognitive model, which posits that developing awareness of and control over one’s own cognitive processes is a key cognitive resource for complex problem-solving outcomes (Flavell, 1979).

Another important contribution of this study is the finding that the relationship between mathematical metacognition and computational thinking is partially explained through cognitive flexibility. The findings suggest that metacognitive awareness may be associated with individuals’ ability to shift between strategies and generate alternative solution pathways during complex problem solving. In this context, mathematical metacognition appears to be associated with more adaptive forms of computational thinking through greater flexibility in cognitive strategy use (Badolo et al., 2025; Schoenfeld, 2013, 2014; Zohar and Barzilai, 2013). These findings are consistent with previous research indicating that metacognitive regulation and cognitive flexibility are closely associated in demanding cognitive tasks (Diamond, 2013; Schraw and Dennison, 1994). On the other hand, some studies argue that the relationship between mathematical metacognition and advanced thinking skills operates largely through direct pathways (Zohar and Barzilai, 2013). What distinguishes the present study from these approaches is its demonstration that this relationship also functions through a regulatory mechanism such as cognitive flexibility. This difference may be attributable to the use of an adolescent high school sample in which executive functions and cognitive flexibility are still maturing, making the indirect effects of metacognitive regulation on computational thinking more apparent (Igazság et al., 2019; Poon, 2018). Accordingly, the findings suggest that the relationship between mathematical metacognition and computational thinking is not unidimensional process, but rather a multilayered structure that operates through executive function mechanisms such as cognitive flexibility.

These findings help establish a conceptual link between Flavell’s theory of metacognition and Wing’s framework of computational thinking (Flavell, 1979; Wing, 2006, 2011). Within this framework, mathematical metacognition appears to be associated with individuals’ capacity to monitor and regulate cognitive processes, whereas cognitive flexibility may support the adaptive use of computational thinking strategies during complex problem solving. Accordingly, cognitive flexibility may be understood as a functional mechanism linking metacognitive regulation with computational thinking.

Another important finding of the study is that the relationship between cognitive flexibility and computational thinking varies depending on grade level. The observed interaction effects and simple slope analyses indicate that the positive association between cognitive flexibility and computational thinking is stronger at higher grade levels. This pattern suggests that the influence of these cognitive variables may vary according to students’ academic and developmental experiences. Research conducted in different countries suggests that increasing curriculum intensity may be associated with greater reliance on cognitive flexibility (Best and Miller, 2010; Diamond, 2013; OECD, 2019). Similarly, studies conducted in the Turkish context indicate that upper-grade students, who face greater academic pressure and tasks requiring multi-step problem solving, may rely on a broader repertoire of cognitive strategies and activate their cognitive flexibility more effectively (Karslı, 2019). Within this framework, the stronger association between cognitive flexibility and computational thinking at higher grade levels is closely related to the structural demands of the education system. Although cognitive flexibility remains important for computational thinking at lower grade levels, the relatively weaker association may reflect students’ more limited problem-solving experience and narrower strategy repertoires at these stages (Badolo et al., 2025; Honra and Monterola, 2026; Ramos-Salazar and Hayward, 2019). Collectively, the findings suggest that computational thinking is shaped by the interaction of metacognitive awareness, cognitive flexibility, and students’ educational experiences across adolescence. From this perspective, the study contributes to ongoing discussions concerning the cognitive and educational foundations of computational thinking.

### Theoretical and practical implications

The findings of this study offer important contributions to theoretical discussions on the development of computational thinking. First, the partial explanation of the effect of mathematical metacognition on computational thinking through cognitive flexibility demonstrates that metacognitive regulation processes are closely linked to individuals’ capacity for cognitive adaptability. This result extends Flavell’s metacognitive model to help explain the cognitive foundations of computational thinking: metacognition is conceptualized not merely as a structure limited to monitoring and controlling one’s own cognition, but as a higher-order regulatory mechanism that enables cognitive processes to be used in flexible, adaptive, and context-sensitive ways.

In addition, the finding that the relationship between cognitive flexibility and computational thinking becomes more pronounced across higher grade levels aligns with theoretical perspectives emphasizing the developmental nature of computational thinking. While Wing’s computational thinking framework largely focuses on the structural features of cognitive processes, the present study demonstrates that these skills cannot be considered independent of developmental context. The results indicate that computational thinking is not merely a product of individual cognitive abilities, but emerges in conjunction with students’ academic level, learning demands, and the nature of their problem-solving experiences.

The findings also offer important implications for educational practice. In particular, the strengthening of the effect of cognitive flexibility on computational thinking at higher grade levels indicates the need to deliberately support this skill within instructional processes. In mathematics and problem-solving–oriented courses at the high school level, it becomes increasingly important to adopt instructional approaches that encourage students to generate multiple solution paths, compare alternative strategies, and learn from their mistakes. Such learning environments can support cognitive flexibility and, in turn, contribute to the more functional development of computational thinking.

Moreover, the findings highlight the need to design curricula in a way that is sensitive to grade level. Given that students’ cognitive flexibility is still developing at lower grade levels, instructional processes at these stages may benefit from more structured guidance and explicit strategy instruction. In contrast, at higher grade levels, learning activities that are more open-ended, multi-step, and grounded in real-life problems can encourage the active use of cognitive flexibility, helping to move computational thinking beyond a purely technical skill. This study also yields important implications for teacher education and professional development programs. Increasing teachers’ awareness of the relationships among mathematical metacognition, cognitive flexibility, and computational thinking can enhance the quality of classroom practices. When teachers evaluate students’ problem-solving processes in a process-oriented rather than outcome-focused manner and provide metacognitive feedback, they may help foster learning environments associated with the development of both cognitive flexibility and computational thinking skills.

### Limitations and future directions

This study has several limitations, and the findings should be interpreted within these constraints. First, the cross-sectional design limits the ability to draw causal conclusions about the relationships among mathematical metacognition, cognitive flexibility, and computational thinking. Although the proposed moderated mediation model is grounded in theory, longitudinal or experimental research designs are needed to more clearly identify the directionality of these relationships over time.

Second, the sample consisted of high school students from a specific region of Türkiye, which may limit the generalizability of the findings. The sample was drawn exclusively from one city in southeastern Türkiye, which differs from other Turkish regions in terms of socioeconomic profile, academic infrastructure, and student demographics. Given that Turkish secondary education is strongly oriented toward high-stakes national examinations, particularly in the upper grades (Bars, 2026), the moderating pattern observed here may be partly shaped by this examination culture rather than by developmental processes alone. In educational systems with different curricular emphases, pedagogical philosophies, or levels of technology integration, the grade-level moderation pattern may manifest differently or be attenuated (Kovan and Gülbahçe, 2026). Therefore, the findings should be interpreted with caution when applied to samples from other regions or education systems.

Third, all data were collected through self-report measures, which introduces potential biases such as social desirability effects and common method variance. This limitation is especially salient for the computational thinking scale, where self-reported perceptions of competence may diverge from actual performance capabilities. Individuals with limited metacognitive awareness may systematically underestimate their computational thinking competencies, while those with strong metacognitive skills may rate themselves more accurately a pattern that could, in principle, inflate the observed metacognition–CT correlation. Future research incorporating objective, task-based CT assessments alongside self-report instruments would help clarify the extent to which this measurement approach affects the observed associations.

Fourth, while cognitive flexibility was examined as a mediating variable and grade level as a moderating variable, other cognitive and affective factors such as motivation, academic self-efficacy, and learning strategies were not included in the model. This point is underscored by recent work demonstrating that self-efficacy serves as a meaningful mediating mechanism in educational outcomes across different learning contexts (Kholifah et al., 2025). Including self-efficacy as a covariate or additional mediator in future models would enable researchers to better isolate the unique contributions of mathematical metacognition and cognitive flexibility to computational thinking. Future research can build on the relationships identified in this study in several ways. Longitudinal studies could provide stronger evidence for the developmental trajectories of mathematical metacognition and cognitive flexibility. Experimental studies could directly test the effects of metacognitive- and cognitive flexibility–based interventions on computational thinking. Beyond grade level, future studies may examine the moderating roles of gender, academic track, school type, or instructional approach. Besides, computational thinking should be investigated within collaborative problem-solving, group work, and technology-supported learning environments (Saad and Zainudin, 2024). Finally, comparative studies across different cultural settings could clarify whether the relationships between mathematical metacognition and computational thinking are universal or context-specific.

## Conclusion

This study examined the associations among mathematical metacognition, cognitive flexibility, and computational thinking in high school students, with particular attention to the mediating role of cognitive flexibility and the moderating role of grade level. Grounded in Flavell’s metacognitive framework and Wing’s conceptualization of computational thinking, the findings indicate that mathematical metacognition is associated with computational thinking both directly and indirectly through cognitive flexibility. Moreover, the strength of this indirect association varies across grade levels, highlighting the developmental sensitivity of these cognitive processes. The findings further suggest that cognitive flexibility may play a stronger role in the association between metacognitive regulation and computational thinking at higher grade levels, highlighting the context-dependent nature of higher-order thinking skills during adolescence. These findings contribute to current understanding of computational thinking by situating it within a broader metacognitive and developmental framework. Overall, the findings highlight the importance of fostering metacognitive awareness and cognitive flexibility in secondary education to support computational thinking development.

## Data Availability

The raw data supporting the conclusions of this article will be made available by the authors, without undue reservation.
